# [Corrigendum] Macrophage-secreted IL-8 induces epithelial-mesenchymal transition in hepatocellular carcinoma cells by activating the JAK2/STAT3/Snail pathway

**DOI:** 10.3892/ijo.2024.5672

**Published:** 2024-07-22

**Authors:** Xiu-Tao Fu, Zhi Dai, Kang Song, Zhuo-Jun Zhang, Zheng-Jun Zhou, Shao-Lai Zhou, Yi-Ming Zhao, Yong-Sheng Xiao, Qi-Man Sun, Zhen-Bin Ding, Jia Fan

Int J Oncol 46: 587-596, 2015; DOI: 10.3892/ijo.2014.2761

Subsequently to the publication of the above article, an interested reader drew to the authors' attention that a possible error had been identified in the selection of images in Figs. 1 and/or 7. After having consulted their original data, the authors realized that an erroneous image appeared on p. 593, in Fig. 7F [the 'Hep-G2 / IL-8 (5 ng/ml)' data panel], where part of this figure panel was overlapping with an image on p. 589 in Fig. 1C (the 'Hep-G2 Co-cultured' data panel).

After a thorough review and verification of the data by all the authors, they have confirmed that the original data presented in the paper were accurate, and the error was solely due to the selection of an incorrect image during figure arrangement. The authors confirm that this mistake in image selection did not affect the overall conclusions reported in the article. A corrected version of Fig. 7, including the correct data for the 'Hep-G2 / IL-8 (5 ng/ml)' panel in Fig. 7F, is shown on the next page.

The authors are grateful to the Editor of *International Journal of Oncology* for granting them the opportunity to publish this Corrigendum. All the authors agree to the publication of this Corrigendum, and apologize to the readership for any inconvenience caused.

## Figures and Tables

**Figure 7:**
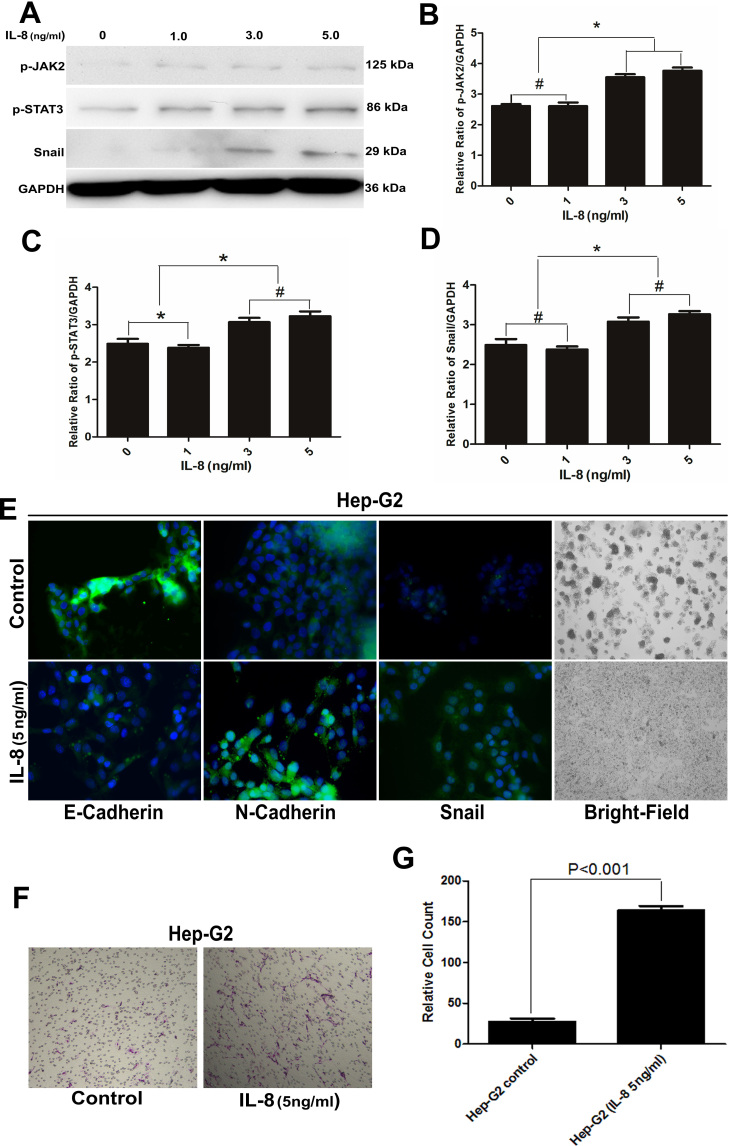
Role of IL-8 signaling in EMT. (A) The expression of Jak2, p-Jak2, Stat3, p-Stat3 and Snail were assessed in Hep-G2 exposure to different concentrations of IL-8. (B-D) Semi-quantitative analysis demonstrated that Jak2/Stat3/Snail pathway could be activated by IL-8. (^#^P>0.05; ^*^P<0.05). (E) Immunofluorescent analysis of E-Cadherin, N-Cadherin and Snail in Hep-G2 vs. Hep-G2 that was incubated with IL-8. The green signal represents the staining of the corresponding protein and the blue signal represents the DAPI-stained nuclei. The morphological change of Hep-G2 after incubated with IL-8 was also observed. (F and G) Functional analysis is illustrated after IL-8 had been added to the media for 24 h. A Transwell assay showed that the number of invading Hep-G2 cells were higher that in control groups (original magnification, ×100).

